# Separation of Isomeric Tau Phosphopeptides from Alzheimer’s Disease Brain by Cyclic Ion Mobility Mass Spectrometry

**DOI:** 10.1021/jasms.2c00289

**Published:** 2023-01-27

**Authors:** Andrej Kováč, Petra Majerová, Marianna Nytka, Monika Zajacová Cechová, Petr Bednář, Roman Hájek, Dale A. Cooper-Shepherd, Alexander Muck, Karel Lemr

**Affiliations:** † Institute of Neuroimmunology, Slovak Academy of Sciences, Dúbravská cesta 9, 845 10 Bratislava, Slovak Republic; ‡ Axon Neuroscience R&D Services SE, Dvořákovo nábrežie 10, 811 02 Bratislava, Slovak Republic; § Department of Analytical Chemistry, Faculty of Science, Palacky University, 17 listopadu 12, 771 46 Olomouc, Czech Republic; ∥ Waters Corporation, Stamford Avenue, Altrincham Road, Wilmslow SK9 4AX, United Kingdom; ⊥ Institute of Microbiology of the Czech Academy of Sciences, Vídeňská 1083, 142 20 Prague, Czech Republic

## Abstract

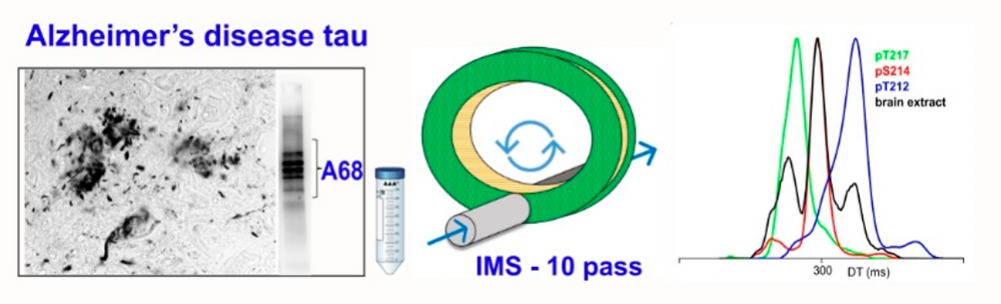

Alzheimer’s disease (AD) is a neurodegenerative disorder of increasing concern. It belongs to diseases termed tauopathies which are characterized by inclusions of abnormally hyperphosphorylated and truncated forms of the protein tau. Studies of tauopathies often focus on detection and characterization of these aberrant tau proteoforms, in particular the phosphorylation sites, which represent a significant analytical challenge for example when several phosphosites can be present on the same peptide. Such isomers can even be difficult to fully separate chromatographically. Since recently introduced cyclic ion mobility–mass spectrometry can offer different selectivity, we have investigated the closely positioned phosphorylation sites S214, T212, and T217 of a tryptic peptide from proline rich region of tau–TPSLPTPPTREPK. The conformational heterogeneity of the isomeric peptides in the gas phase hindered their separation due to their overlapping arrival time distributions. Increasing the resolution of the analysis alone is insufficient to distinguish the peptides in a mixture typical of patient samples. We therefore developed a method based on a combination of collision-induced dissociation, isomeric product ions (*m*/*z* 677) mobility separation and post-mobility dissociation to aid in analyzing the isomeric phosphopeptides of tau in diseased brain extract. For all three isomers (T212, S214, and T217), the ion mobility signal of the ion at *m*/*z* 677 was still observable at the concentration of 0.1 nmol/L. This work not only offers insights into the phosphorylation of tau protein in AD but also provides an analytical workflow for the characterization of challenging pathological protein modifications in neurodegenerative diseases.

## Introduction

Tauopathies are sporadic or familial neurodegenerative disorders characterized by intracellular inclusions of abnormal hyperphosphorylated and truncated tau protein.^1^ Tauopathies involve around 20 different neurodegenerative diseases including the most frequent tauopathy—Alzheimer’s disease (AD). Under physiological conditions, tau protein is a soluble intracellular protein whose function is to control the stability of the axonal microtubules. In tauopathies, tau protein is abnormally modified, resulting in reduced affinity for microtubules and disruptions of the cytoskeleton. They aggregate into intracellular oligomers, paired helical filaments, and neurofibrillary tangles in neuronal or glial cells.^2^ Hyperphosphorylated tau proteins are observed in neurofibrillary pathological lesions. The altered phosphorylation results from dysregulation of the kinases and phosphatases that modulate the tau phosphoproteome. Extensive analysis of the human AD brain has shown the presence of many hyperphosphorylated tau sites, which promote tau aggregation and the formation of neurofibrillary tangles.^3^ Several phosphorylated residues are significantly enriched in the cerebrospinal fluid (CSF) of AD patients.^4^ The exact mechanism or the critical phosphorylation sites are not yet known. However, qualitative and quantitative characterization of tau protein phosphorylation is important to describe the changes during aggregation of insoluble tau throughout disease progression. The longest full-length tau isoform consists of 441 amino acids and has 85 potential phosphorylation sites (5 tyrosines, 35 threonines, and 45 serines). Several phosphorylations in the proline rich region of tau have been quantified in CSF using commercially available in vitro diagnostics (IVD) immunoassay kits discriminating AD patients, mild cognitive impairment (MCI), and healthy subjects. Recent studies using ultrasensitive immunoassays have shown that plasma tau is a valid progression biomarker in AD.^5^ Although immunoassays have high specificity and sensitivity, they exhibit substantial disadvantages especially for quantification of heavily phosphorylated regions of tau. Liquid chromatography-tandem mass spectrometry (LC–MS/MS) based workflows have been therefore used to quantify individual tau phosphopeptides. McAvoy et al.^6^ developed the first quantitative method for analysis of tau in CSF by mass spectrometry. Barthelemy et al. proposed a method for monitoring tau peptides in CSF using liquid chromatography-high resolution mass spectrometry.^7^ Recently, this approach was applied to an analysis of CSF from AD patients at mild to moderate stages and non-AD subjects. Higher tau phosphorylation rates in AD patients were observed. The degree of hyperphosphorylation was higher for T111, T205, S208, and T217, than for T181 which is usually employed as a biomarker for AD.^8^ Phosphorylation on threonine 217 was identified in brain tissue, CSF, and plasma of patients and is considered as a new potential biomarker for AD.^9,10^ An LC–MS analysis of two phosphorylated tau isoforms (pT181 and pT217) has been recently reported supporting that pT217 can be a useful biomarker of Alzheimer’s disease.^11^ In sarkosyl insoluble fraction of brain tissue, different phosphorylations were also confirmed by mass spectrometry.^12^

The complexity of analyzed samples encourages researchers to take advantage of new technical designs. The incorporation of orthogonal separation techniques such as high-resolution ion mobility spectrometry (HRIM) coupled to mass spectrometry can complement the LC separation monitoring post-translational modifications. Ion mobility based on structure for lossless ion manipulation (SLIM) resolved isobaric peptides coeluting in reversed-phase liquid chromatography.^13^ Synthetic phosphopeptides were analyzed using field asymmetric waveform ion mobility (FAIMS)^14^ and drift tube ion mobility (DTIMS)^15^ in the form of 3+ and 2+ ions. DTIMS (resolving power ∼80–100) separated isomeric phosphopeptides less completely than FAIMS as the conformers of phosphopeptides were partly overlapped. However, DTIMS still enabled isomers’ recognition and offered higher sensitivity. Phosphorylation sites were localized for peptides even at the concentration of 0.5 nmol/L. FAIMS also differentiated synthetic phosphopeptides related to human tau-protein with modification at adjacent sites. The resolving power 140–190 was achieved using a residence time of 0.2 s. The separation was better for 3+ and 4+ ions than 2+.^16^

Cyclic IM (cIM) has recently been implemented in a research-grade high-resolution Q-TOF mass spectrometer. The traditional linear ion mobility region is replaced with a compact circular traveling-wave ion mobility cell. Ions traverse around the cyclic device filled with nitrogen as an inert buffer gas and with every pass, greater ion mobility resolution is achieved. The cyclic device provides scalable, high-resolution ion mobility separations and introduces the unique ability to perform ion mobility/ion mobility (IM^*n*^) experiments, extending the benefits of ion mobility separation.^17^

To resolve the complexity of tau-phosphorylation sites, we have used the cIM instrumental platform for the first time for analysis of specific phosphorylated sites on a tau protein isolated from AD brain. Advanced multistage collisional dissociation experiments in combination with cyclic ion mobility enabled the distinction of important phosphorylated tau peptide isomers with limited sample preparation and without chromatographic separation. The proposed method represents the methodological advance in the field of AD research.

## Materials and Methods

### Sample Preparation

The lyophilized modified peptides TPpSLPTPPTREPK (S214), pTPSLPTPPTREPK (T212), TPSLPpTPPTREPK (T217) (ThermoFischer Scientific, Waltham, U.S.A.) were stored at −80 °C until dilution. The stock solutions were prepared at 1 μmol/L concentrations by vortexing at 800 rpm for 1 min at room temperature in a solvent containing 0.1% LC–MS grade formic acid in 50% aqueous LC–MS acetonitrile (both from Sigma-Aldrich, Saint-Louis, U.S.A.). The peptides were then diluted to 100 nmol/L working concentration in the same solvent. Testing the detection, lower concentrations were used (1 nmol/L, 0.1 nmol/L).

### Human Brain Tissue

Alzheimer’s disease brain tissue (Braak stage VI) was obtained from Slovak Brain Bank (51-45739480-A0001, SK010922). All experiments were monitored and approved by the Institutional Ethics Committee (Ethics Committee of Institute of Neuroimmunology, Slovak Academy of Sciences).

### Extraction of Tau Proteins from Human Brain

Human brain tissue (1 g) was homogenized in 5 mL of TTL lysis buffer (50 mM Tris-HCl pH 7.4, 150 mM NaCl, 1 mM EDTA, 0.5% Triton X-100, 1 mM Na_3_VO_4_, 20 mM NaF, 1 × protease inhibitors complete EDTA-free, all from Sigma-Aldrich, Saint-Louis, U.S.A.) using an OMNI TH homogenizer (Omni International, Kennesaw, U.S.A.) at medium speed for 2 min at 4 °C. The homogenate was centrifuged at 30 000*g* for 20 min at 4 °C. The supernatant was transferred into a fresh tube. The pellet was homogenized in the same volume of TTL lysis buffer and under the same conditions. After centrifugation, both supernatants were pooled.

### Immunoaffinity Purification of Tau Protein

The sample was diluted 2-fold with cold WBNP0.1 buffer (50 mM Tris-HCl pH 7.4, 1 M NaCl, 0.1% NP-40, all from Sigma-Aldrich, Saint-Louis, U.S.A.) at 4 °C and centrifuged at 30 000*g* for 20 min at 4 °C. The supernatant was passed through a 0.2 μm filter. Tau proteins were isolated from the brain tissue by affinity purification using an antitau antibody cartridge. The affinity column was prepared by covalent coupling of DC18 antibody (aa 168–181, specific for human tau protein, from AXON Neuroscience SE, Bratislava, Slovakia) to CNBr-activated Sepharose 4 Fast Flow (GE Healthcare, Chicago, U.S.A.). After the supernatant was passed through the DC18 column, the eluate was collected. The column was washed as follows: 4 × 2 mL of WBNP0.1, 4 × 2 mL of WBNP1 (50 mM Tris-HCl pH 7.4, 1 M NaCl, 0.1% NP-40), 4 × 2 mL of WBNP0.1, 4 × 2 mL of wash buffer, and once with 2 mL of 100 mmol/L ammonium bicarbonate. Bound tau proteins were eluted with 800 μL of 200 mmol/L formic acid. Elution was repeated three times to ensure the complete elution of bound proteins. Finally, the eluate was dried using a SpeedVac vacuum concentrator.

### Biochemical Western Blot Analysis

Samples were separated into 12% SDS-polyacrylamide gels and transferred to a nitrocellulose membrane in 10 mM *N*-cyclohexyl-3-aminopropanesulfonic acid (CAPS, pH 11, Roth, Karlsruhe, Germany). The membranes were blocked in 5% milk in Tris-buffered saline with 0.1% Tween 20 (Sigma-Aldrich, St. Louis, U.S.A.) (TBS-T, 137 mM NaCl, 20 mM Tris-base, pH 7.4, 0.1% Tween 20) for 1 h and incubated with primary antibody overnight at 4 °C. As phospho-dependent antitau antibodies, we used: antihuman DC217 (1:50, Axon Neuroscience R&D SE, Bratislava, Slovakia) and monoclonal antihuman pThr212 (1:1000, Invitrogen Life Technologies, Carlsbad, U.S.A.). For total tau, we used an antihuman DC190 antibody (recognizing epitope 368–376, 1:50, Axon Neuroscience R&D SE, Bratislava, Slovakia). Membranes were incubated with horseradish peroxidase (HRP)-conjugated secondary antibody in TBS-T (1:3000, Dako, Glostrup, Denmark) for 1 h at RT. Immunoreactive proteins were detected by chemiluminescence (SuperSignal West Pico Chemiluminescent Substrate, Thermo Scientific, Pittsburgh, U.S.A.) and the signals were digitized by Image Reader LAS-3000 (FUJIFILM, Bratislava, Slovakia) (Figure S1 of the Supporting Information, SI).

### Trypsin Digestion of Tau Proteins

The dried eluate was dissolved in 100 μL of Tris- HCl, pH 8 supplemented with 1 μL of 1 mol/L dithiothreitol (DTT, Sigma-Aldrich, Saint-Louis, U.S.A.) and incubated for 60 min at 37 °C. After reduction, the sample was supplemented with 3 μL of 500 mmol/L iodoacetamide (Sigma-Aldrich, Saint-Louis, U.S.A.) to a final concentration 15 mmol/L. The alkylation was performed in the dark for 30 min at 25 °C. One ng of trypsin and 1 mmol/L CaCl_2_ were added to the sample and incubated overnight at 37 °C. Digested tau proteins were dried in a SpeedVac.

### Mass Spectrometry

The samples were introduced into the cIM instrument by direct infusion to a standard flow ESI source (Waters Corporation, Wilmslow, U.K.). The instrument design was discussed in detail elsewhere.^17^ Briefly, the instrument encompasses a new generation hybrid Q-IM-TOF geometry with a cyclic ion mobility separator. Ions are transferred from the source through the first vacuum stages using stacked-rf-ring ion guides (StepWave) which propel ions toward the quadrupole mass filter. The subsequent trapping cell is used for accumulating ions before IM separation, and also functions as a collision cell. The resulting ion packets are then transported through an ion guide (IG) and injected into a helium cell. The subsequent ion guide transports the ions into a multifunctional array of pin electrodes (“T-Wave Array”). The programmable character of this multifunctional “switch” is the key part of the functionality of this instrument in combination with a cyclic arrangement of the IM cell (Figure S2). The cIM cell is filled with nitrogen to a pressure of ∼1.8 mbar. The traveling wave (T-Wave) height propagating the ion separation was set to 15 V, the traveling wave velocity to 375 m/s, data were acquired as two TOF pushes per one data bin, ADC detector start delay was offset automatically (for example, 12 ms single pass, 55 ms five pass experiments). The offset corresponds to the sum of injection time (the injection of ions to the multifunctional array) and separate time (the multifunctional array drives ions to the cyclic drift cell). During this period, ions may not yet appear on a detector. The sequences of various experiments are fully accessible and programmable in the software user interface (Advanced Cyclic Editor) through the instrument tune page. The specific timing sequences used for IM selection are described in the Discussion section. For post-IM peptide fragmentation, a transfer cell collision voltage of 25 V was used (transfer CE). The orthogonal acceleration time-of-flight (oa-ToF) featured an offset V or W flight path geometry allowing *m*/*z* measurements at predefined resolutions of >60 000 and 100 000 fwhm, respectively. In this work, we have used the “V” mode for peptide acquisitions.

### Data Analysis

Data were analyzed using Masslynx v.4.2 (Software Change Note 1016), and a modified version of Driftscope v.2.9 (both Waters Corporation, Wilmslow, U.K.). The software records mass spectral data in the final step when ions exit IM cell (“eject and acquire step”). For cyclic separation, the injection or reinjection times (each 10 ms) were not subtracted. Mobilograms were smoothed by mean averaging of 2 points in 1 cycle if not stated otherwise. Fragment spectra were analyzed after summing up the IM peak width at 10% from the base. The spectra were annotated for theoretical fragments of the modified peptide sequence using BioLynx Protein Editor (Waters Corporation, Wilmslow, U.K.) or by ProSight Lite v1.4 (Kelleher Research Group, Northwestern University, Evanston, U.S.A.).

## Results and Discussion

### Single and Multipass Ion Mobility Experiments

**Figure 1:**
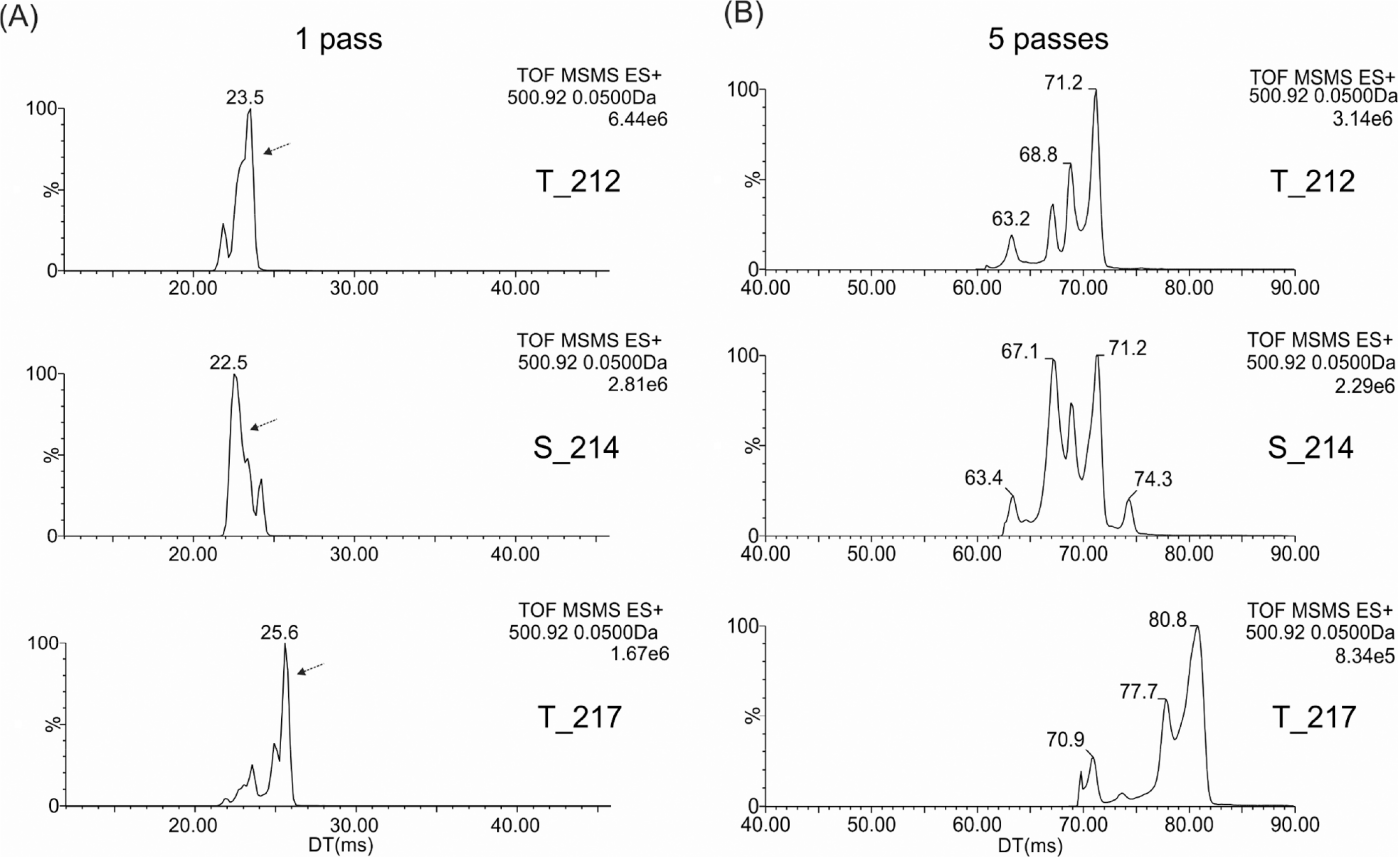
Ion mobilograms of [M+3H]^3+^ (*m*/*z* 500.92) after its isolation in a quadrupole (Q-IMS-TOF experiment).

The sequence TPSLPTPPTREPK of the studied peptide resides in the proline rich region of tau. Its hyperphosphorylation on serine 214, threonine 212, threonine 217, and possibly 220 has been proven to be associated with AD.^20,22^ Because of the phosphorylation on either of these sites produces isomers, we have employed the new highly resolving cIM technique for their differentiation and the identification of individual phosphorylation sites. First, we investigated the protonated forms and mobility behavior of individual synthetic peptides. In the full scan mass spectra of standards (Figure S3A), the predominant peptide signals were attributed to the doubly- and triply protonated molecules at *m*/*z* 750.88 and 500.92, respectively, with evidence of some background ions. The [M+3H]^3+^ and [M+2H]^2+^ mobility peaks of S214, T212, and T217 appeared around 23 and 29 ms. These ions and the background components could be readily differentiated in related single pass total ion mobilograms (Figure S3B). Multipass cyclic ion mobility was focused on both [M+3H]^3+^ and [M+2H]^2+^ to investigate the separation of the isomeric peptides. The triply protonated molecules displayed rather heterogeneous arrival time distributions with several minor shoulders in single pass separation (Figure 1A). After performing five passes of cyclic ion mobility (Figure 1B), peptides exhibit more major conformational states or protomeric isomers; four for T212 (61–73 ms), five for S214 (62–76 ms), and a minimum of four states for T217 (69–83 ms). This large number of gas phase ion peaks, corresponding to either various protonation states and/or conformational assemblies, is related to the structural diversity of the studied peptides enabling different charge localization and shape stabilization. In fact, T217 appears to be well separated from both T212 and S214 but its minor species overlap significantly with the latter two peptides. So, while the conformations of the individual peptides show good separation from each other, the arrival time range over which they separate is similar for all three peptides, meaning complete separation of the peptides into discrete mobility windows in a mixture is not possible. This creates a barrier to accurately distinguishing the peptides akin to poor chromatographic resolution.

As the cIM resolving power (*R*) has been characterized as *R* = *R*_1_*(number of cycles)^0.5^ (ref (17)), *R* can increase from *R*_1_ (cca 65) in 1 pass to ∼2.2*R*_1_ in 5 passes and up to ∼3.2*R*_1_ in 10 passes. The increased resolving power in this case led to increased distance and resolution of individual conformers/protomeric isomers for triply charged molecules. However, a mixture of three phosphopeptides would “move together” resulting in overlapping and convoluted ATDs.

**Figure 2:**
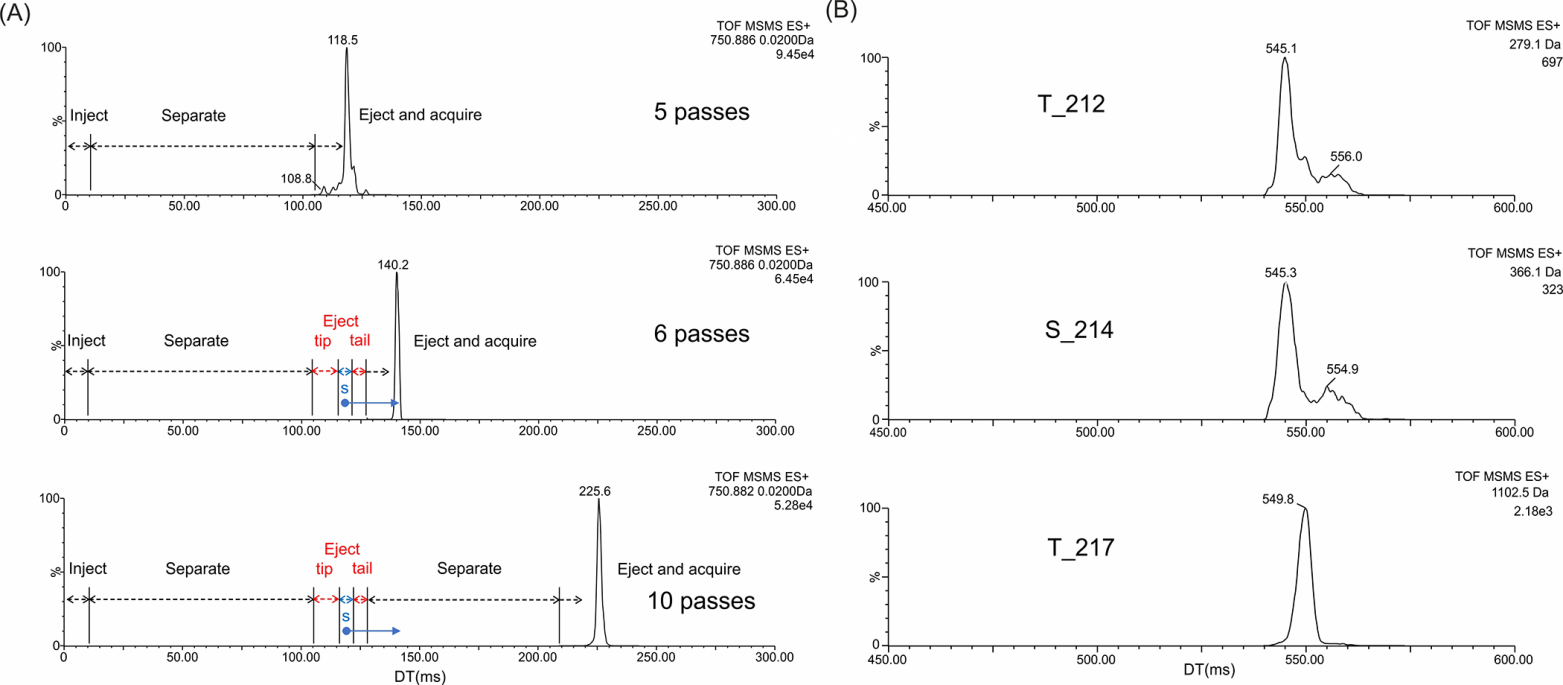
Extracted ion mobilograms of [M+2H]^2+^ of peptide mixture (A) demonstrating tip and tail experiment with multipass separation. Five pass separation before isolation is followed by further 1 or 5 passes of major peak slice (s) in the cyclic IM cell. Q-IMS-TOF of 2+ charge state after 25 pass separation of major peak slice and CID in transfer (26 V) (B). Extracted ion mobilograms of ion b2 at *m*/*z* 279.1 for T212, b3 at *m*/*z* 366.1 for S214 and T212, and y9 at *m*/*z* 1102.5 for T217.

The peptide ions [M+2H]^2+^ exhibited a more homogeneous arrival time distribution with one major peak (compare Figures 1 and S4). The IM2 selection has been employed to this charge state. The first separate step in the sequence was followed by removing the left and right flank of the peak. The sequence employed was therefore as follows: inject → separate for 5 passes → eject front (tip) → isolate major peak slice (s) → eject tail → separate major peak slice for the desired number of further passes → eject and acquire, schematically displayed in Figure 2A (“6 passes, 10 passes”). Currently, as there is no specific terminology accepted by the broader community, this experiment was described as “tip and tail with multipass separation”. This approach enables the separation of only the desired protomeric isomers and conformers from a complex mixture. Individual peptides contribute to the mixture by lower number of conformers (ideally by one) and the overlap of ATD profiles of individual peptides might be eliminated. By performing a tip and tail experiment on the major ion population of the 2+ charge state from the peptide mixture with a total of 25 passes of separation we observed only separation of the major T217 conformer from the major conformers of other two phosphorylation forms T212 and S214 that, however, still overlap. This is shown by the extracted ion mobilograms (EIMs) of characteristic ions (N terminal ions b2 at *m*/*z* 279.1 for T212, b3 at *m*/*z* 366.1 for both S214 and T212, and C terminal y9 ion at *m*/*z* 1102.5 for T217) (Figure 2B). Since ATD profiles of doubly charged peptides still overlap and triply charged molecules exhibit higher signal intensity (Figure S3A), they were used further and finally employed to analyze the brain tissue (see below).

Although, all studied peptides at the protonated molecule level (either doubly or triply protonated) were not completely separated, the described avenues represent available approaches for phosphorylation studies based on sequence and position of modification. While not all ways are closed for distinguishing the precursor ions (other adducts could be investigated for example), we focused on the third option, that is, separation at the product ion level. As the product ions are smaller in size there is the potential for the presence of the phosphorylation to induce a greater proportional shape change in the product ions relative to the precursor ions. The phosphosites on the three peptides are very close to the N-termini, meaning the b ion series will also be isomeric past residue six (that is, corresponding to the phosphosite of T217). This raises the question of using product ions without mobility separation for detection of these peptides, but observed characteristic fragment ions are few, particularly due to the proximity of the modifications. Only one unique ion was found in the spectra (*m*/*z* 1102.5 for T217). Other potentially useful ions (Table S1) are common to the two studied phosphopeptides but they differ in their intensities (half order and more). Nevertheless, the difference in intensity may not be sufficient to discriminate isomeric phosphopeptides in their mixture. In general, the contents of peptides can differ, and a major one can overlap a minor one. It underlines the importance of mobility separation. To test the utility of product ion separation we performed quadrupole selection of the [M+3H]^3+^ and subjected it to collision-induced dissociation in the trap (prior to ion mobility). The resulting product ions were then subjected to multipass ion mobility separation to see if the isomeric product ions could be distinguished.

**Figure 3:**
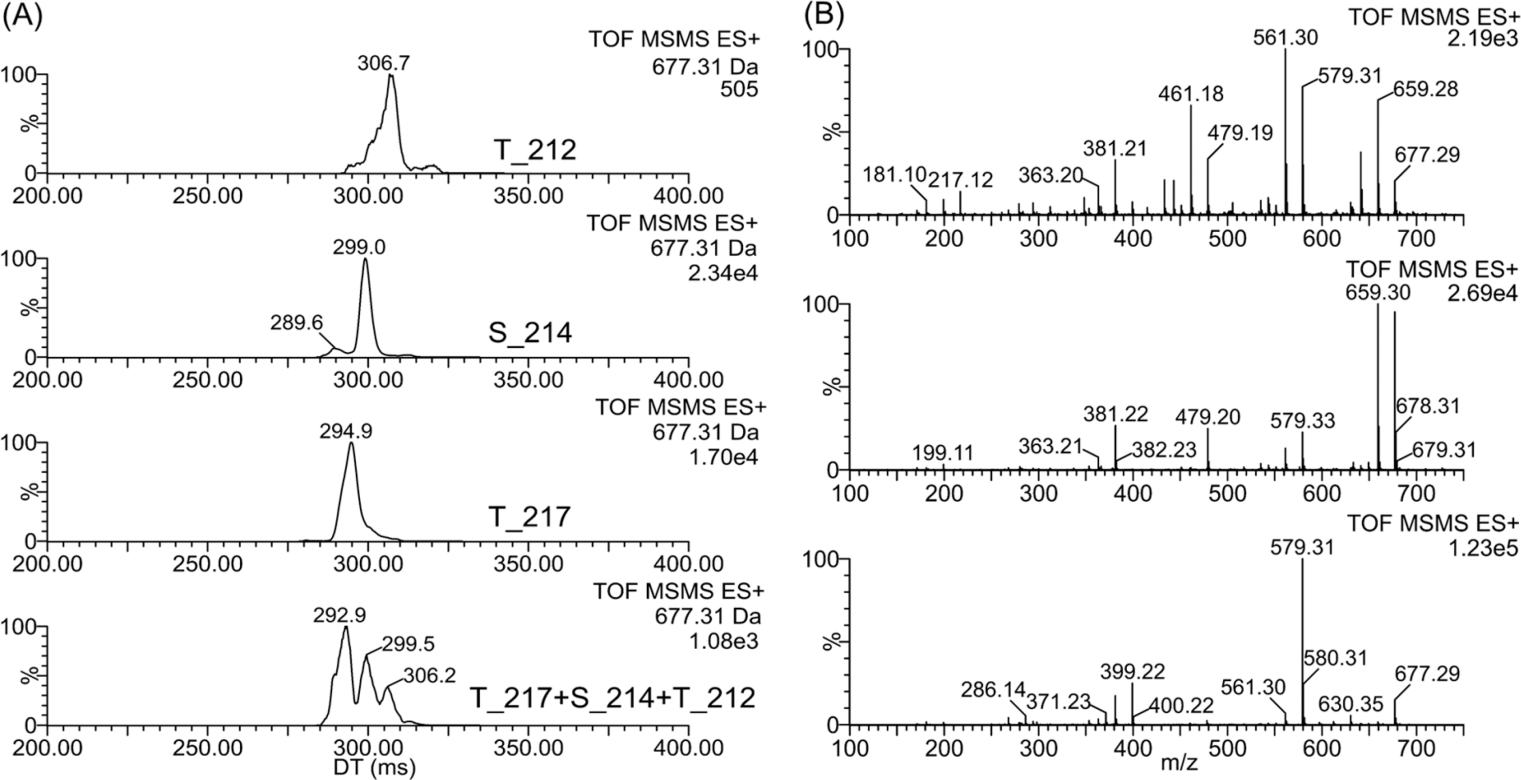
Extracted ion mobilograms of N terminal b6 fragment ion at *m*/*z* 677 (A) generated by CID in trap from [M+3H]^3+^ for T212, S214, T217 (25 V), and their mixture (24 V). Fragment spectra of *m*/*z* 677 obtained by CID after ion mobility separation (10 passes) in transfer (25 V) for individual peptides T212, S214, and T217 (B, top to bottom).

Indeed, the separation of all three phosphopeptides was achieved by a 10 pass cIM experiment for the N-terminal b6 fragment ion at *m*/*z* 677 (herein called a MS2-CID-HRIM experiment). The peaks’ maxima were well separated, and the presence of individual peptides in the mixture could be detected, although signals still overlapped partially (Figure 3A). CID in transfer (after ion mobility) provides fragment spectra of the ion at *m*/*z* 677 (Figure 3B) showing differences that can contribute to peptides’ distinguishing. Using 10 pass experiment, the ion mobility signals of the isomeric ions at *m*/*z* 677 were visible at the concentration of 1 nmol/L, and even 0.1 nmol/L (Figure S5), which is well comparable with the earlier reported localization of phosphorylation sites by the drift tube (0.5 nmol/L).^14^

### Analysis of Brain Tissue from Alzheimer’s Disease

**Figure 4:**
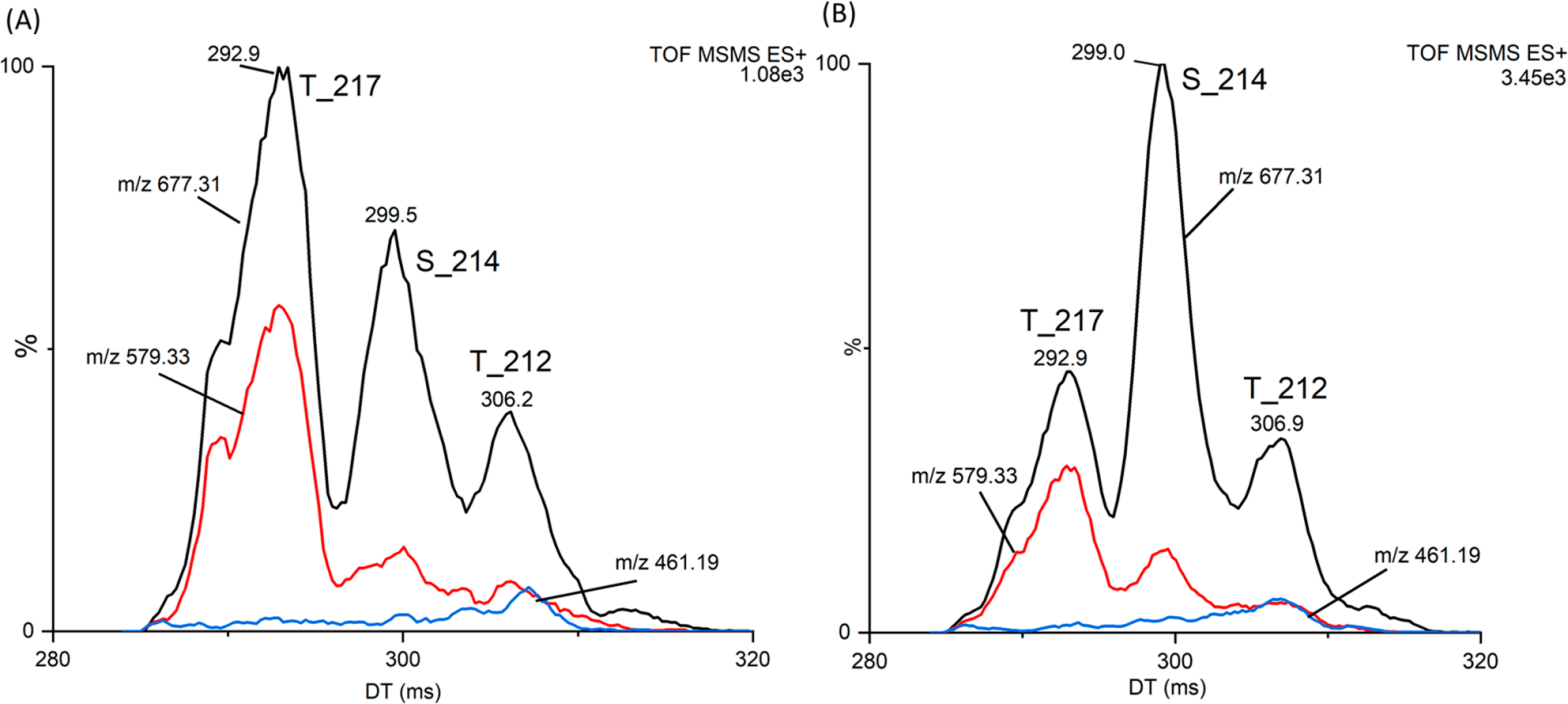
Ion mobility analysis of AD brain extract in comparison to a standard mixture. Extracted ion mobilograms for fragment ion *m*/*z* 677 and its fragment ions at *m*/*z* 579 and 461 showing relative intensities characteristic of individual peptides: mixture of the standards in molar ratio 1:1:1(A), brain extract (B). Ion at *m*/*z* 677 was generated by CID in trap (24 V) from [M+3H]^3+^ ion selected in quadrupole, and was fragmented after ion mobility separation (10 passes) in transfer (25 V).

The method developed using phosphorylated standards was applied to the immunoaffinity purified tau from AD brain tissue. The efficiency of brain tissue’s extraction was confirmed by Western blot analysis (Figure S1). The *MS2-CID-HRIM* (10 passes) of the b6 fragment ion at *m*/*z* 677 showed distinct mobility peaks with clear and reproducible maxima at 293, 299, and 307 ms (Figure 4B), respectively, corresponding well to those for T217, S214, and T212 standards (compare Figure 4A and 4B). The number of IM passes was kept at 10 as ion current may have suffered at significantly higher resolution. To demonstrate the selectivity of our method we performed the *MS2-CID-HRIM* experiment with additional subsequent post-IM CID in the transfer cell, termed *MS2-CID-HRIM-CID*. Again, the [M+3H]^3+^ precursor at *m*/*z* 500.9 from the brain extract was isolated with the quadrupole (*MS2*) and subjected to fragmentation in the trap (*CID*) to generate a series of product ions including the b6 ion at *m*/*z* 677. Multipass IM (10 passes) was performed on this ion (*HRIM*) which was then further fragmented in the transfer collision cell (CID after ion mobility separation). Similarly to [M+3H]^3+^ (Table S1), the ion at *m*/*z* 677 did not provide observable fragment ions that would be unique to individual isomeric phosphopeptides. It underlines the importance of ion mobility separation. We plotted the extracted ion mobilograms for *m*/*z* 677, its fragment ions *m*/*z* 579 (phosphoric acid neutral loss), and 461 (b4-H_2_O) as their relative intensities are characteristic of individual phosphopeptides. Since these ions are not unique to individual isomers, the phosphopeptides’ detection in their mixture solely using CID of mass-selected precursors can fail, but CID after ion mobility supports the detection of separated phosphopeptides. EIMs of the ions demonstrate good agreement between standards and phosphopeptides detected in a brain tissue (Figure 4). It might suggest the higher level of the S214 in AD brain extract. Our results, although not absolutely quantitative are in good agreement with biochemical studies on human AD brain tissue extracts which showed robust phosphosporylation of tau at the S214 epitope.^18^

As shown previously, the specific phosphorylation sites might establish alternative biomarkers of tauopathies.^8,19^ The analysis of phosphoproteome changes in tauopathies is, therefore, an important area of AD research.^20^ Moreover, finding new techniques of detection and quantification of the selectively phosphorylated residues on different isoforms of tau protein in biological fluids for diagnostic purposes is essential for the development of effective treatments.^21^

## Conclusions

Here we reported that high resolution IM–MS is an effective technique for distinguishing isomeric tau phosphopeptides that is applicable to the analysis of human biological samples. We were able to distinguish three isomeric phosphosites using a combination of experiments culminating in a *MS2-CID-HRIM* method, that is, by a 10 pass ion mobility separation of N-terminal b6 fragment ions at *m*/*z* 677 generated from [M+3H]^3+^ prior to ion mobility separation. The selectivity of the method has been confirmed by further post-IM CID experiments (*MS2-CID-HRIM-CID*) providing ion fragments of *m*/*z* 677 with relative intensities characteristic of individual phosphopeptides. The combination of ion mobility separation and two steps fragmentation (in front and behind an ion mobility device) has significantly enhanced the selectivity of the analytical methods. With this method, all three isomeric phosphorylated peptides were detected in human AD brain extract. In combination with a good separation technique and proper sample preparation this method could be used in the future for detailed studies of tau proteome in body fluids that are essential for diagnosis of AD.

## Supporting Information Available

Figure S1. Western blot analysis of brain extract; Figure S2. Schematic of the Waters Select Series Cyclic IM Spectrometer; Figure S3. Separation of studied tau phosphopeptides by cIM; Figure S4. Ion mobilograms of [M+2H]^2+^ after 5 pass separation; Figure S5. 10 pass ion mobilograms of phosphopeptides at low concentrations; Table S1. Characteristic fragment ions for precursor [M+3H]^3+^ (PDF)
