# Supplementary material for: Separation of Isomeric Tau Phosphopeptides from Alzheimer’s Disease Brain by Cyclic Ion Mobility Mass Spectrometry
Source: J Am Soc Mass Spectrom. 2023 Jan 27;34(3):394–400. doi: 10.1021/jasms.2c00289 (PMC10017020; doi:10.1021/jasms.2c00289)
Supplement: Supplementary file 1 — js2c00289_si_001.pdf [file js2c00289_si_001.pdf]

Supporting Information

Separation of isomeric tau phosphopeptides from Alzheimer's disease brain by cyclic ion mobility mass spectrometry

Andrej Kováč[1], Petra Majerová[2], Marianna Nytka[3], Monika Zajacová Cechová[3], Petr Bednář[3], Roman Hájek[4], Dale A. Cooper-Shepherd[4], Alexander Muck[4], Karel Lemr[3,5*]

[1]Institute of Neuroimmunology, Slovak Academy of Sciences, Dúbravská cesta 9, 845 10, Bratislava, Slovak Republic

[2]Axon Neuroscience R&D Services SE, Dvořákovo nábrežie 10, 811 02 Bratislava, Slovak Republic

[3]Department of Analytical Chemistry, Faculty of Science, Palacky University, 17.listopadu 12, 771 46 Olomouc, Czech Republic

[4]Waters Corporation, Stamford Avenue, Altrincham Road, Wilmslow, SK9 4AX, U.K.

[5]Institute of Microbiology of the Czech Academy of Sciences, Vídeňská 1083, 142 20 Prague, Czech Republic

[*]Corresponding author, e-mail: karel.lemr@upol.cz

Table of contents

Figure S1. Western blot analysis of brain extract

Figure S2. Schematic of the Waters Select Series Cyclic IM Spectrometer

Figure S3. Separation of studied tau phosphopeptides by cIM

Figure S4. Ion mobilograms of $[M+2H]^{2+}$ after 5 pass separation

Figure S5. Ten pass ion mobilograms of phosphopeptides at low concentrations

Table S1. Characteristic fragment ions for precursor $[M+3H]^{3+}$

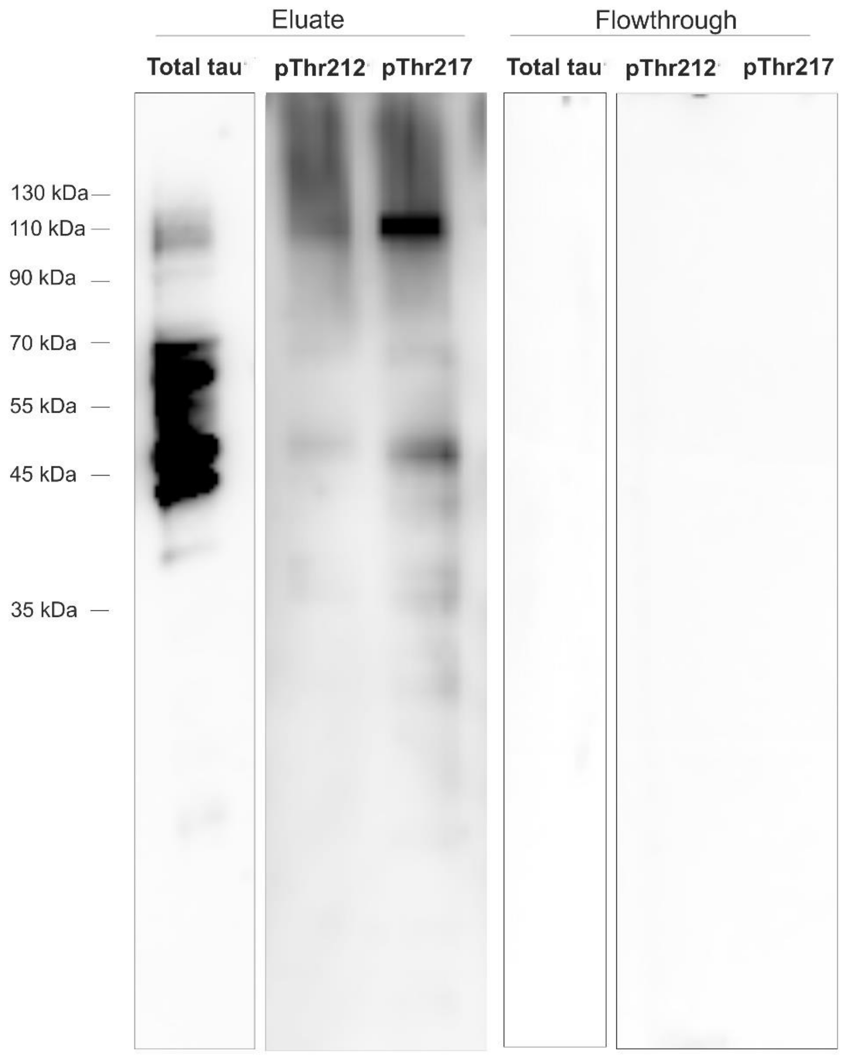

Figure S1. Western blot analysis of brain extract. The AD brains eluates after immuno-pulldown were analysed by Western blot using phosphorylation-dependent anti-tau antibodies against pThr212 and pThr217. Total tau was determined with a DC190 anti-tau antibody.

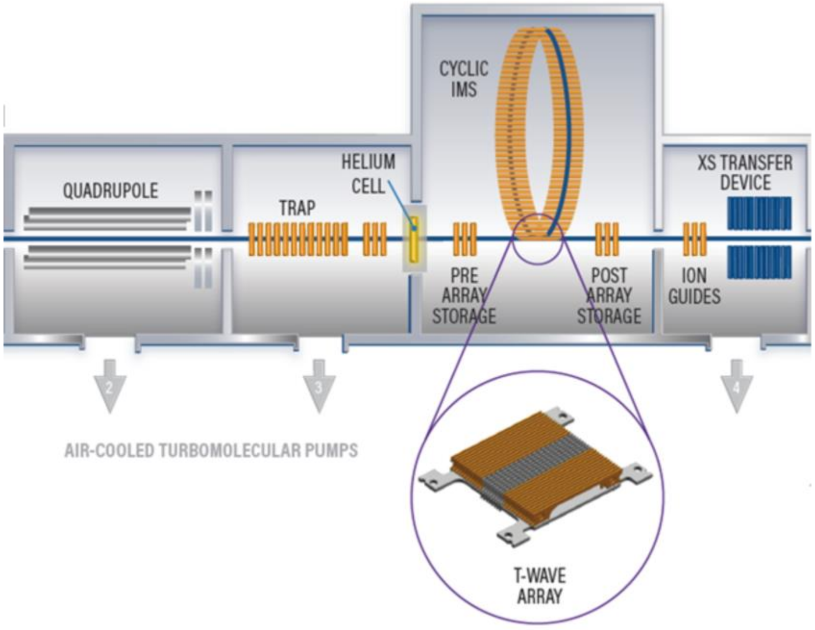

Figure S2. Schematic of the Waters Select Series Cyclic IM Spectrometer. After the initial trapping of ions in the first collision cell (trap), they are injected into the multifunctional array (T-wave array) where they are accelerated sidewise by a travelling wave into the cyclic separation IM cell.[1] Reprinted in part with permission of co-author Dale A. Cooper-Shepherd from "Application of cyclic ion mobility coupled to mass spectrometry for high peak capacity analysis of native and deuterated peptide mixtures", Martin Palmer; Malcolm Anderson; Dale A. Cooper-Shepherd; James I. Langridge; Robert Tonge; John R. Engen, poster nr. ThP285, Proceedings of 67th ASMS Conference on Mass Spectrometry and Allied Topics, Atlanta, Georgia, U.S.A., 2019. Copyright 2019 Waters Corporation.

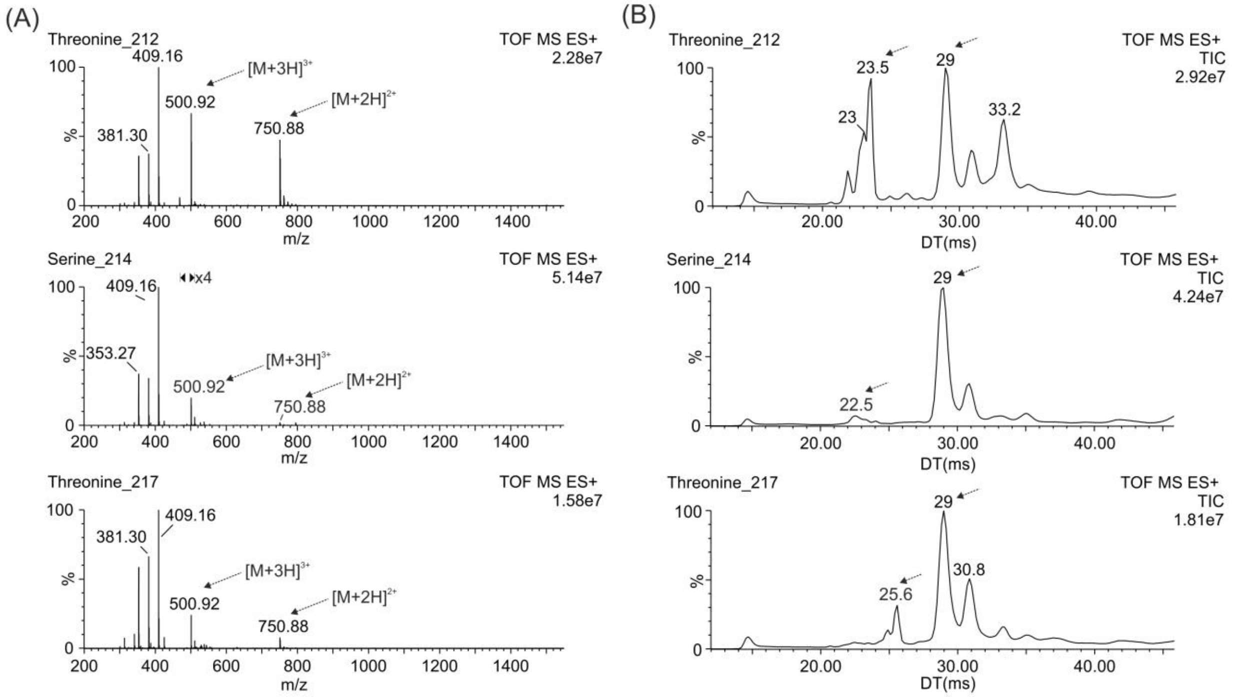

Figure S3. Separation of studied tau phosphopeptides by cIM. The full scan MS spectra (A) and single pass total ion mobilograms (B) of infused standards of isomeric phosphopeptides (T212, S214 and T217). Arrows mark mobility peaks related to $[M+3H]^{3+}$ (around 23 ms) and $[M+2H]^{2+}$ (cca 29 ms).

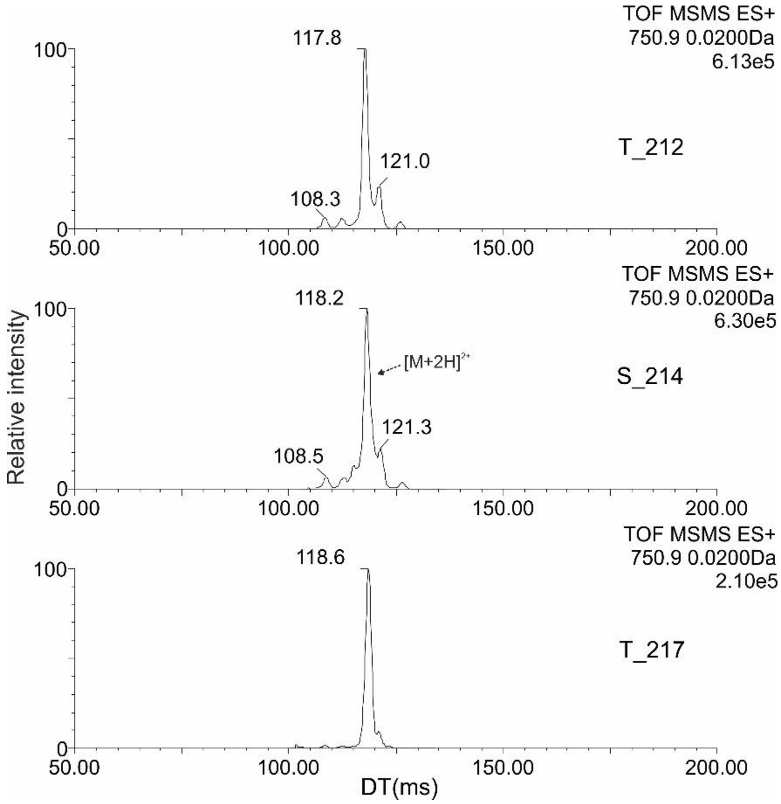

Figure S4. Ion mobilograms of $[M+2H]^{2+}$ after 5 pass separation. Extracted ion mobilograms of precursor ion at m/z 750.9.

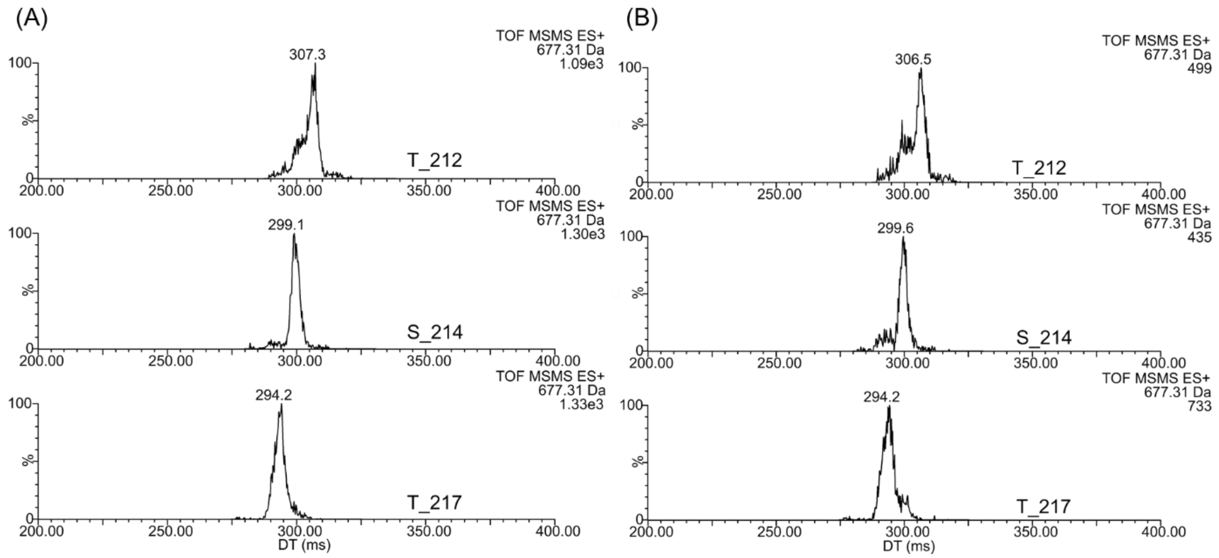

Figure S5. Ten pass ion mobilograms of phosphopeptides at low concentrations. Extracted ion mobilograms of N terminal b6 fragment ion at m/z 677 generated by CID in trap (24 V) from $[M+3H]^{3+}$ for T212, S214, T217, the concentration of standards: 1 nmol/L (A); 0.1 nmol/L (B); without smoothing, drift time aligned to Figure 3 by subtracting 4 ms.

Table S1. Characteristic fragment ions for precursor $[M+3H]^{3+}$. A unique fragment ion is in bold; for common fragment ions differing significantly in signal intensity, s stands for stronger, w for weaker.

phosphopeptides	fragment ions (m/z)
**T212**	279.1 (s); 286.1 (w); 1116.6 (s); 1135.7 (s); 1222.7 (s)
**S214**	
**T217**	279.1 (w); 286.1 (s); **1102.5**; 1116.6 (w); 1135.7 (w); 1222.7 (w)

Reference

(1) Giles, K.; Ujma, J.; Wildgoose, J.; Pringle, S.; Richardson, K.; Langridge, D.; Green, M. A Cyclic Ion Mobility-Mass Spectrometry System. *Anal. Chem.* **2019**, *91* (13), 8564-8573.
